# Enhancing prognosis in multiple myeloma bone disease: insights from a retrospective analysis of surgical interventions

**DOI:** 10.3389/fsurg.2024.1433265

**Published:** 2024-12-19

**Authors:** Xiangjun Shi, Xingchen Yao, Yue Wu, Boran Du, Xinru Du

**Affiliations:** ^1^ Beijing Tiantan Hospital, Capital Medical University, Beijing, China; ^2^ Department of Orthopedics, Beijing Chaoyang Hospital, Capital Medical University, Beijing, China; ^3^ Department of Pharmacy, Capital Medical University, Beijing Obstetrics and Gynecology Hospital, Beijing, China

**Keywords:** multiple myeloma, prognosis, surgical, multiple myeloma bone disease, chemotherapy

## Abstract

**Background:**

Multiple myeloma (MM) is a hematological malignancy characterized by bone marrow infiltration and osteolytic tumor formation. Despite advancements in the treatment of this disease, MM remains incurable and often leads to complications, such as multiple myeloma bone disease (MMBD). Surgical intervention is frequently necessary to manage symptoms associated with bone disease, including pain and fractures.

**Methods:**

A retrospective review was conducted on 135 patients diagnosed with MMBD who had undergone surgery, compared to 190 patients diagnosed with MM who had not undergone surgery and served as controls. Surgical interventions were performed based on typical clinical presentations of myeloma-related bone disease, as indicated by imaging results. Patients who had only undergone percutaneous kyphoplasty or vertebroplasty (PKP/PVP) were excluded from this study.

**Results:**

Among patients who underwent surgery, the spine was the most common site of bone metastasis, accounting for 50% of cases. The number of operations (overall survival [OS], *p* = 0.82; progression-free survival [PS], *p* = 0.41) and the order of surgery and chemotherapy treatment (OS, *p* = 0.85; PS, *p* = 0.83) did not significantly impact the outcomes. Further, MM patients with surgery exhibited a significant prognostic difference compared to those without surgery (OS, *p* < 0.0001). The International Staging System (ISS) stage serves as a prognostic factor for MMBD who have undergone surgery, with higher ISS stages indicating worse prognoses.

**Conclusions:**

These results indicate that surgery and chemotherapy together improved patient survival rates compared to chemotherapy alone, thereby facilitating patients' acceptance of systemic chemotherapy. Furthermore, the appropriate timing of surgery contributes to the positive prognoses of patients with MMBD.

## Introduction

Multiple myeloma (MM) is an intractable hematological malignancy of plasma cells that often infiltrate the bone marrow and form osteolytic tumors. In recent years, the survival period of patients with MM has been significantly prolonged; however MM is not completely curable. The prolonged survival period has caused the clinical incidence of various MM complications to increase every year (1, 2). The basis of treatment for patients with MM include bone marrow stem cell suppression and immune machine targeted therapy; the main drugs used include bisphosphonates, protease inhibitors, immunomodulators and monoclonal antibodies (3). Multiple myeloma bone disease (MMBD) is a serious complication of MM (4). A cardinal clinical feature of MM is the presence of osteolytic bone lesions (OBD) (5–7), which are accompanied by bone pain, increased risk of fracture and tumor-induced hypercalcemia. OBD affects 80% of patients with MM with a negative impact on both quality of life and overall survival (8). The bone is a common site of cancer spread, with various common cancers, including MM, breast cancer prostate cancer and lung cancer, which are reported to cause bone destruction (9). The majority of patients with MM can be treated for intractable pain with chemotherapy and radiotherapy (10). In patients with bone disease symptoms, such as bone marrow and nerve compression and large soft tissue masses, the advantages of chemotherapy and radiotherapy are limited, and surgery is often required (11, 12). The purpose of surgical treatment is not to cure MM, but rather to treat the related osteolytic lesions via surgical intervention (13). Surgery can improve the quality of life, reduce pain and suffering and prolong survival. Surgical options include vertebral body reconstruction, arthroplasty and vertebroplasty which can address pathological fractures of the long bones of the limbs and their associated pain and dysfunction. Successful surgical treatment can effectively relieve pain restore the continuity interrupted by fractures, aid the restoration of limb function and improve the quality of life (14, 15). Surgical methods involve resection or curettage of the lesion, filling the defect with bone cement and the application of one of various internal fixation methods according to the context. The spine surgery of the patients with MM includes vertebroplasty/kyphoplasty, while percutaneous kyphoplasty/vertebroplasty (PKP/PVP) is suitable for patients with MM with compression fractures caused by osteolytic destruction of the vertebral body but not accompanied by spinal cord compression. Open spine surgeries have anterior, posterior or combined anterior and posterior approaches. Surgery includes tumor removal and decompression followed by spinal reconstruction and internal fixation (16–18). Quiet et al. (19) confirmed the efficacy and safety of surgery for symptomatic spinal lesions in patients with MM. The primary objectives of surgical resection are to prolong the survival of patients, while secondary objectives include symptom remission and the limitation of tumor progression to improve quality of life and survival (20). The majority of the patients with OBD at diagnosis are treated with surgery and chemotherapy; however, surgery is not suitable for all patients (21).

To date, there have been no large-scale clinical studies evaluating the effectiveness of surgical interventions compared with chemotherapy in prolonging overall survival (OS). To address this problem, the prognoses and clinical and laboratory characteristics of patients with surgical intervention and chemotherapy were compared with those of patients with chemotherapy alone. Factors surrounding surgery that may also affect prognoses were also considered.

## Patients and methods

### Patient selection

Patients who were diagnosed with MM were retrospectively reviewed between September 2018 to February 2020. Patients with missing data were excluded from the analysis. Following approval by the ethics committee, informed written consent that has been blinded for peer review was obtained for all subjects. All diagnosed patients with MM received a traditional chemotherapy regimen, containing thalidomide or bortezomib (22). A total of 325 patients were included of whom 135 that had undergone surgery and 190 had not undergone surgery. Surgery was performed upon clinical presentation of typical myeloma-related bone disease, as evaluated using imaging results. The patients undergoing only PKP/PVP were excluded from the study. We continue to track the survival time of patients after surgery, starting from the time of the initial surgery. After recurrence and subsequent surgery, the time is still calculated from the time of the initial surgery. In our study, surgery is usually recommended for patients with MM for pathological fractures, spinal cord or nerve root compression, or lytic bone lesions.

### Surgical operation and follow-up

Spinal surgery for MM bone disease involved posterior decompression, partial tumor resection, subsequent use of bone cement to fill the lesion defect and pedicle screw fixation decompression, tumor resection and the use of bone cement filling and internal fixation by cage and anterior plate. Long bone surgery involved tumor scraping, fracture reduction, internal fixation using screw and plate system and the use of bone cement to fill the defect in order to reconstruct bone integrity. The follow-up was conducted until death (end point) or until July 2020 and the median follow-up time was 6.5 years (2–12 years). The progression-free survival (PS) and OS were calculated using Kaplan-Meier survival analysis and logarithmic rank test. The period of PS was counted from the day of surgery.

### Statistical analysis

PS and OS were analyzed using the Kaplan-Meier survival estimate. The Cox proportional hazard model was used to compare survival curves. Statistical analyses were conducted using *R* (version 3.5.0) and visualized by survminer package (https://github.com/kassambara/survminer). *P* < 0.05 was considered to indicate a statistically significant difference. The two groups were compared by the *χ*^2^ test and the *t*-test.

## Results

### Clinical characteristics of patients with MM with and without surgery

Out of 325 patients with MM, 135 had undergone surgery and 190 had not. Of the 135 patients wo had undergone surgery, 83 (61%) patients were male and 52 (39%) were female. Of the 190 patients, 103 (54%) patients were male and 87 (46%) were female. No significant difference was noted in the age distributions of surgical and non-surgical groups. Significant differences were noted in the distribution of heavy chain-type MM (Fisher exact test, *P* < 0.05), light chain-type MM (*χ*^2^ = 5.212, *P* < 0.05, Cramer's *V* = 0.132, *P* < 0.05) and international staging system (ISS) stage (*W*^2^ = 7.483, *P* < 0.05, Cramer's *V* = 0.157, *P* < 0.05). No significant difference was noted in the Durie-Salmon stage between the two groups (Fisher's exact test, *P* > 0.05). The clinical features of patients with MM between the two groups are listed in Table 1. No difference was noted in the treatment or treatment intensity between the two groups in chemotherapy. The excluded cases of patient heavy chain information from the two groups include non-secretory cases, those lacking an M component and cases with unclear characteristics.

**Table 1:** Epidemiologic and clinical characteristics of patients of multiple myeloma.

Variable	Category	Number of patients with surgery (percentage)	Number of patients without surgery (percentage)	*P* value
Sex				
	Male	83 (61%)	103 (54%)	*P* > 0.05
	Female	52 (39%)	87 (46%)	*P* > 0.05
Age (years)				
	<60	75 (56%)	88 (46%)	*P* > 0.05
	≥60	60 (44%)	102 (54%)	*P* > 0.05
Heavy chain				*P <* 0.05
	IgG	58 (55%)	88 (59%)	
	IgA	29 (28%)	37 (25%)	
	IgD	3 (3%)	19 (13%)	
	IgM	0	0	
	IgE	0	0	
	No heavy chain	15 (14%)	6 (4%)	
	missing	30 (22%)	40 (21%)	
Light chain				*P <* 0.05
	*λ* light chain	43 (37%)	93 (51%)	
	*κ* light chain	73 (63%)	91 (49%)	
ISS stage				*P <* 0.05
	I	29 (25%)	24 (13%)	
	II	41 (35%)	79 (42%)	
	III	46 (40%)	85 (45%)	
DS stage				*P* > 0.05
	I	4 (3%)	8 (4%)	
	II	13 (11%)	19 (10%)	
	III	103 (86%)	163 (86%)	
ALB (g/dl)	≥35	24 (17%)	43 (23%)	*P* > 0.05
	<35	111 (83%)	147 (77%)	*P* > 0.05
Method of therapy		Surgery + traditional chemotherapy	Traditional therapy	

Data are presented as number (%) or median (range). Difference of variables were tested by Chi-square or Fisher exact test. *P* < 0.05 or *P* < 0.01 was considered statistically significant.

ISS, stage, International Stage System; DS, stage, Durie-Salmon System; ALB, Albumin.

The statistical analysis involved chi-square tests to determine *P* values for categorical variables. Fisher's exact test was employed specifically for categorical variables where expected cell counts were below five. For continuous data, comparisons of mean values were conducted using ANOVA tests.

### Location of lesions

The surgical sites and their frequencies among patients with MMBD are shown in Table 2. The spine was the most common site, accounting for 50% of the lesion frequency. Specifically, the thoracic spine (48 times) was the most common surgical site, followed by the lumbar spine (30 times), cervical spine (5 times) and sacrum (5 times). The surgeries of the upper and lower limbs were 12% and 18%, respectively. The other sites (20%) included the soft tissue (9), rib, breast and shoulder.

**Table 2:** Comparison of lesion detection rate in different bone areas.

	Number	Ratio
Spine	88	50%
Cervical spine	5	
Thoracic spine	48	
Lumbar spine	30	
Sacrum	5	
Upper limbs	22	12%
Clavicle	8	
Humerus	13	
Radius	1	
Lower limbs	32	18%
** **Femoral neck	6	
** **Interchanteric	3	
** **Femoral shaft	19	
** **Tibia	4	
Others	35	20%

135 patients were considered in surgical and accrued 177 times surgery.

### Surgery-improved survival in patients with MM

**Figure 1:**
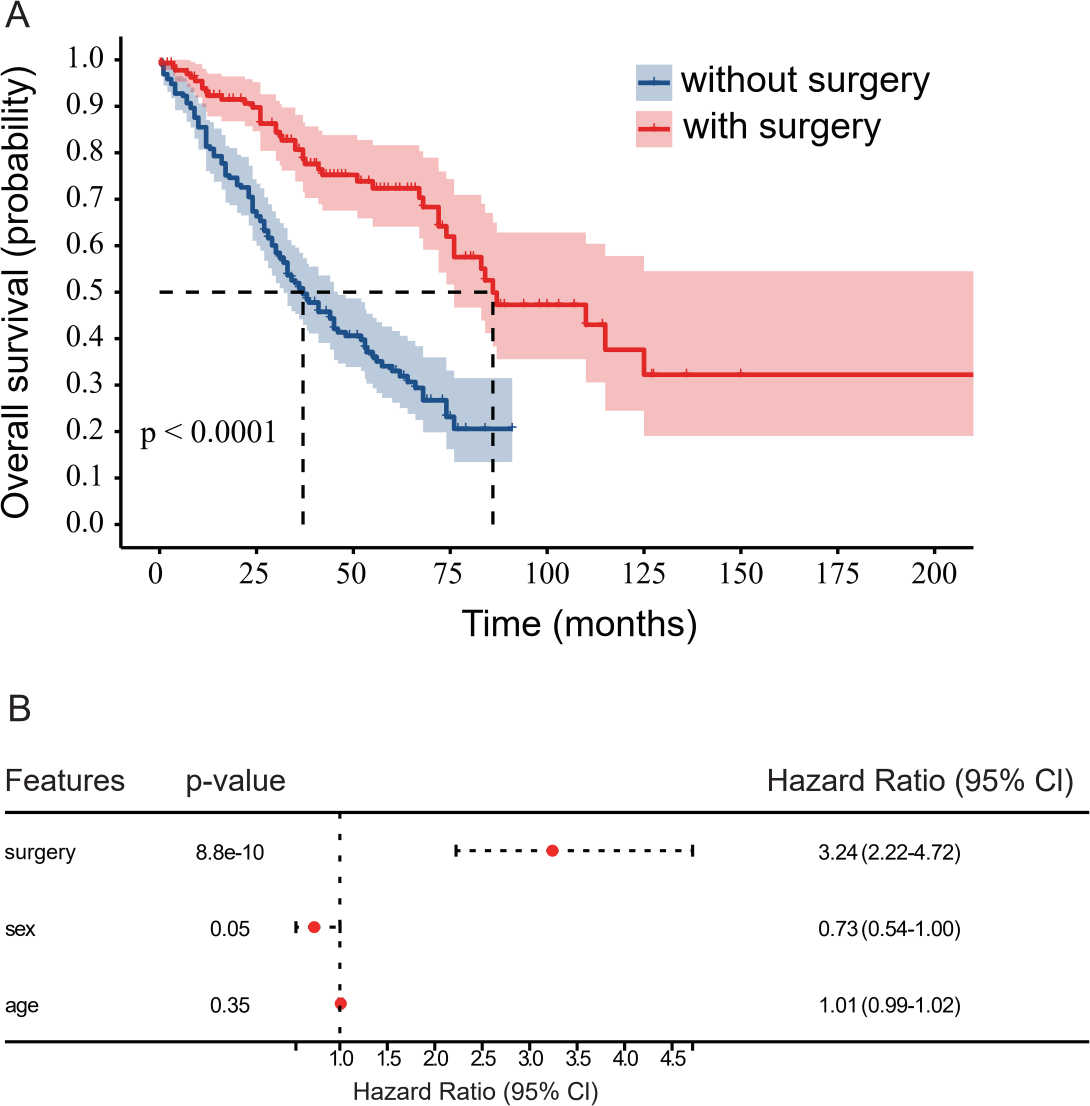
**(A)** Comparison of the prognosis of patients with MM with and without surgery. The OS of patients with MM without surgery was significantly lower than that of patients with MM with surgery (*P* < 0.0001). MM, multiple myeloma; OS, overall survival. **(B)** Multivariate survival analysis conducted using the Cox proportional hazards model.

**Figure 2:**
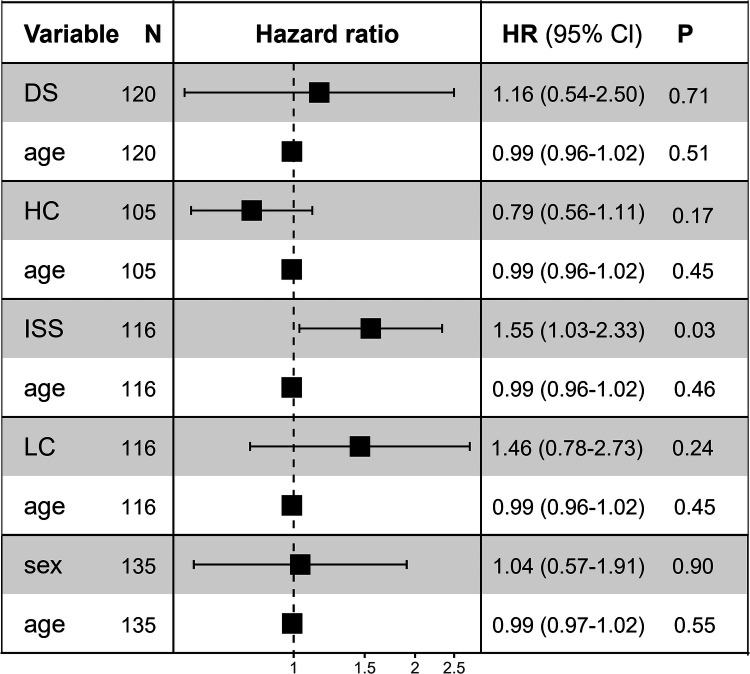
Multivariate Cox regression analysis prognostic factors. HR for disease progression analysis. Squares represent study-specific HR. Horizontal lines indicate 95% CI. The overall heterogeneity of patient age was evaluated using the interaction test and the *P* value is reported. HR, hazard ratio; CI, confidence interval; LC, light chain; HC, heavy chain; ISS, international stage system; DS, Durie-Salmon.

Univariate analysis indicated that the OS rate of patients who underwent surgery was significantly higher than those who did not undergo surgery (*P* < 0.0001). Then we analyzed survival time, survival status, age, gender, and surgical features using the Cox proportional hazards model for multivariate survival analysis, we assessed the prognostic significance of these factors among 325 samples. Our findings showed that patients who underwent surgery had better prognostic outcomes (Figures 1A,B). The median OS time among patients who had undergone surgery was 86 months (0.3–227 months), while that of patients with MM who were only treated with chemotherapy was 37 months (0.5–84 months; Figure 1A). The 5-year survival rate of patients with and without surgery was 31.8% and 17.3%, respectively and the 3-year survival was 58.5% and 44.2%, respectively. Patients with longer survival duration had obtained more benefit from surgical interventions than those with chemotherapy only. Following adjustment for age, multivariate Cox regression analysis of patients with MMBD indicated that ISS was associated with the factors affecting surgery prognosis (*P* < 0.05; Figure 2).

### Lack of significant association of number of patients with surgery and disease prognosis

**Figure 3:**
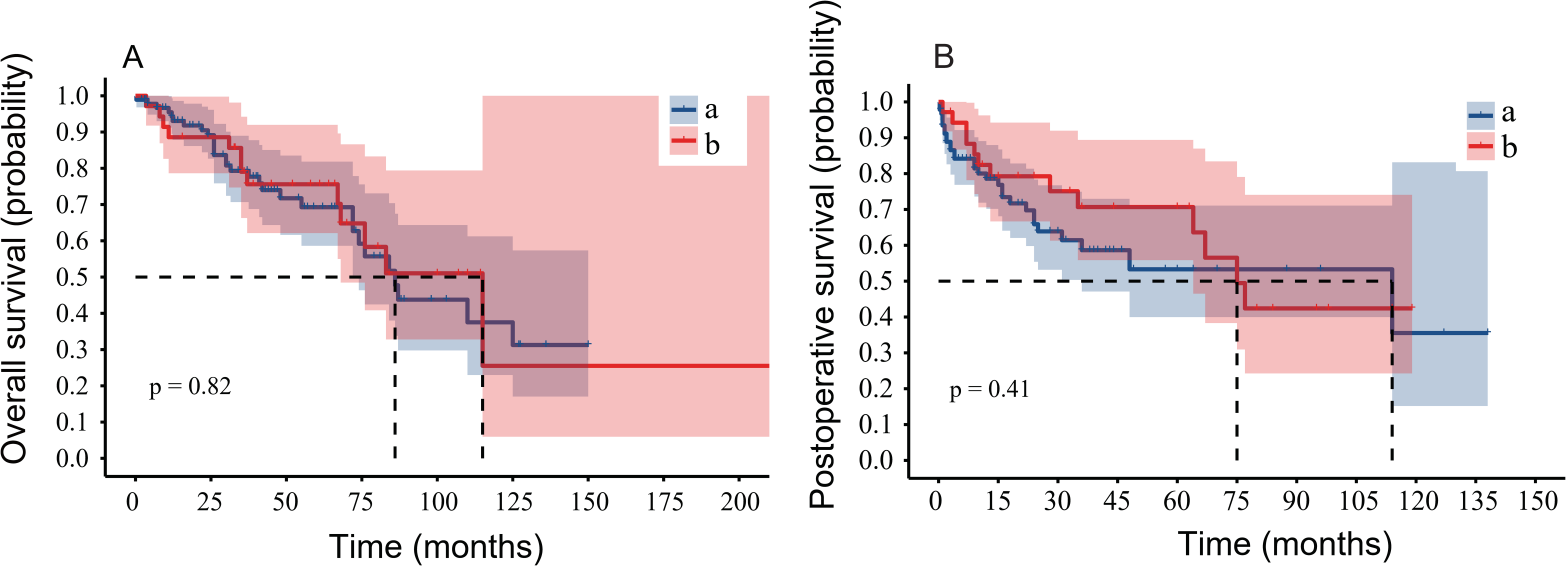
The number of operations is not associated with the prognosis of patients with MMBD. **(A)** The OS of Group A (one time surgery only) and Group B (multiple surgeries) indicated no significant difference (*P* = 0.82). **(B)** The PS between the two groups indicated no significant difference (*P* = 0.41). MMBD, multiple myeloma bone disease; OS, overall survival; PS, progression-free survival.

The present study indicated that the number of surgeries in a single patient was not significantly related to prognosis; this result was noted for all patients with MMBD who underwent surgery. The patients were divided into two groups according to surgery numbers as follows: Group A: One-time surgery and Group B: Two or more surgeries. The median OS of Group A was 86 months (0.3–150 months), while the median OS of Group B was 115 months (3.5–227 months; Figure 3A). The 5-year OS survival rate of patients in groups A and B was 27.1% and 48.5%, respectively and the 3-year OS survival rate was 54.3% and 65.7% for each of these two groups, respectively. However, this difference was not significant (*P* > 0.05). No significant difference was noted in PS between the patients of Groups A and B, whereas the median survival was 114 months (0.3–138 months) and 75 months (1–114 months; Figure 3B), respectively. The 5-year PS survival rate of groups A and B was 7.6% and 37.1%, respectively, whereas the 3-year PS survival rate for each of these two groups was 20.6% and 45.7%, respectively. No significant difference was noted in OS and PS between groups A and B.

### The order of surgery and chemotherapy has no influence on prognosis

**Figure 4:**
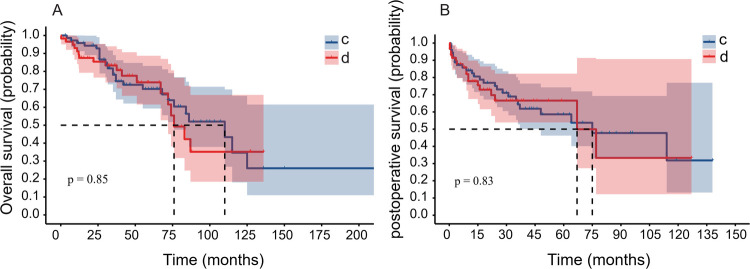
The sequence of surgery timing was not associated with prognosis. **(A)** The OS of Group C (patients with MMBD who received chemotherapy first) and Group D (patients with MMBD who received surgery first) indicated lack of significant difference (*P* = 0.85). **(B)** The PS between the two groups indicated lack of significant difference (*P* = 0.83). OS, overall survival; MMBD, multiple myeloma bone disease; PS, progression-free survival.

The information on the association of the order of surgery and chemotherapy and its effects on disease prognosis is limited. To address this, the surgery timing relative to chemotherapy was assessed and its effect on patient prognosis. Group C included patients with MMBD who received chemotherapy first, followed by surgery, and group D included patients with MMBD who received surgery first, followed by chemotherapy. Group C exhibited a median OS and 5-year OS rate of 110 months (3–227 months) and 38.3%, respectively, while Group D had a median OS and 5-year OS rate of 76 months (0.3–136 months) and 23.7%, respectively. No significant difference was noted between the two groups (*P* > 0.05; Figure 4A). The 3-year OS rates of Groups C and D were 60.2% and 55.9%, respectively. The median PS of Groups C and D were 75 months (0.7–138 months) and 67 months (0.3–127 months), respectively; no significant difference was noted between these two groups (*P* > 0.05; Figure 4B). The 5-year PS rate of Groups C and D were 21.9% and 6.7%, respectively; the 3-year PS rate of Groups C and D were 39.7% and 20.3%, respectively.

### The influence of ISS on survival of patients with MMBD

**Figure 5:**
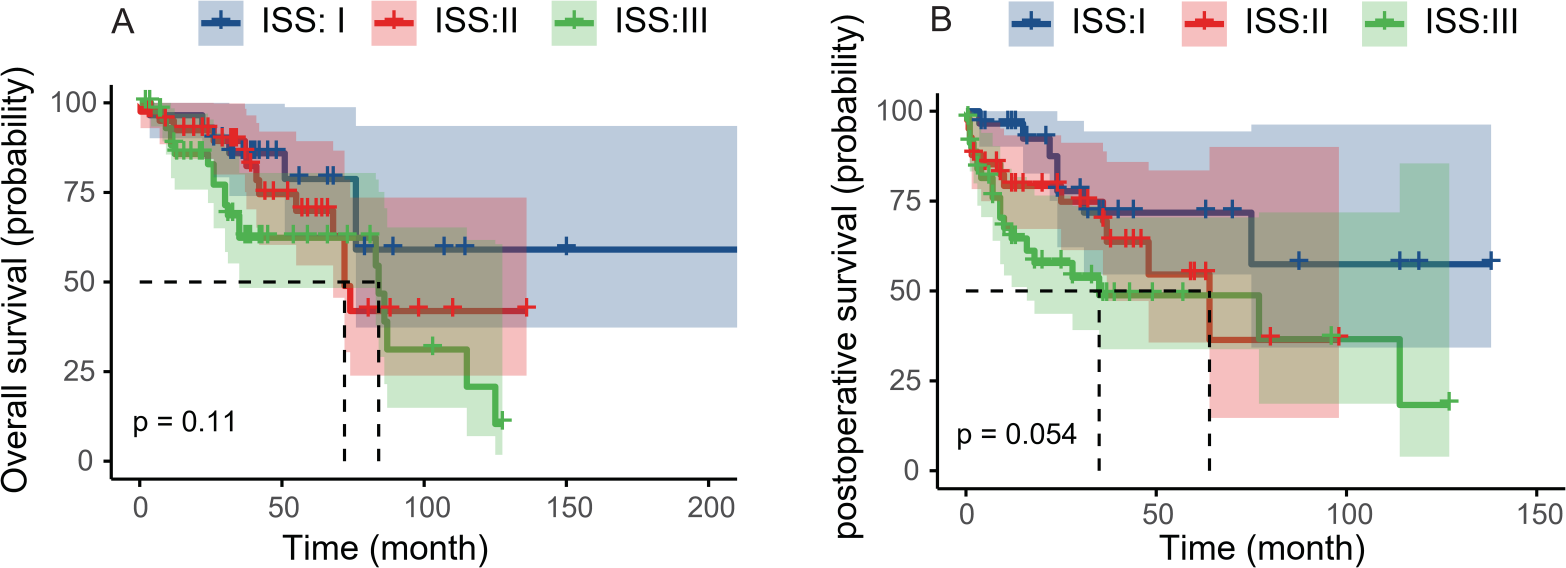
ISS stage III predicts worse prognosis. **(A)** The OS of ISS and ISS II or ISS II and III indicated lack of significant differences (*P* = 0.52, *P* = 0.11). The OS between ISS I and III indicated a significant difference (*P* = 0.04). **(B)** The PS of ISS I and II or ISS II and III indicated lack of significant differences (*P* = 0.20, *P* = 0.054). The PS between ISS I and III indicated a significant difference (*P* = 0.01). ISS, international stage system; OS, overall survival; PS, progression-free survival.

Cox proportional hazard regression analysis was used to identify the predictive factors for MMBD patient survival. Initially, the effect of surgery was examined based on the DS and ISS stages. The majority of patients with MMBD who underwent surgery were DS stage III; the DS stage exhibited no significant influence on prognosis (data not shown). The median OS and 5-year OS rates of patients with ISS II and III were 72 months and 11.1% and 84 months and 9.4%, respectively. The 5-year OS rate of patients with ISS I was 8.5%. No significant difference was noted in the OS of patients with ISS I and II (*P* > 0.05) or between patients with ISS II and III (*P* > 0.05). However, a significant difference was noted in the OS of patients with ISS I and III (*P* < 0.05; Figure 5A). The 3-year OS rates of ISS I, II and III were 7.6%, 12.8% and 22.2%, respectively. The median PS and 5-year PS rates of ISS II and III were 64 months and 3.4% and 35 months and 3.4%, respectively. The 5-year PS rate of ISS I was 6.0%. No significant difference was noted in PS between patients with ISS I and II (*P* > 0.05) or between patients with ISS II and III (*P* > 0.05), while a significant difference was noted in PS between patients with ISS I and III (*P* < 0.05; Figure 5B). The 3-year PS rates of ISS I, II and III were 7.7%, 10.2% and 7.7%, respectively.

### A case study of a patient with three separate surgeries

**Figure 6:**
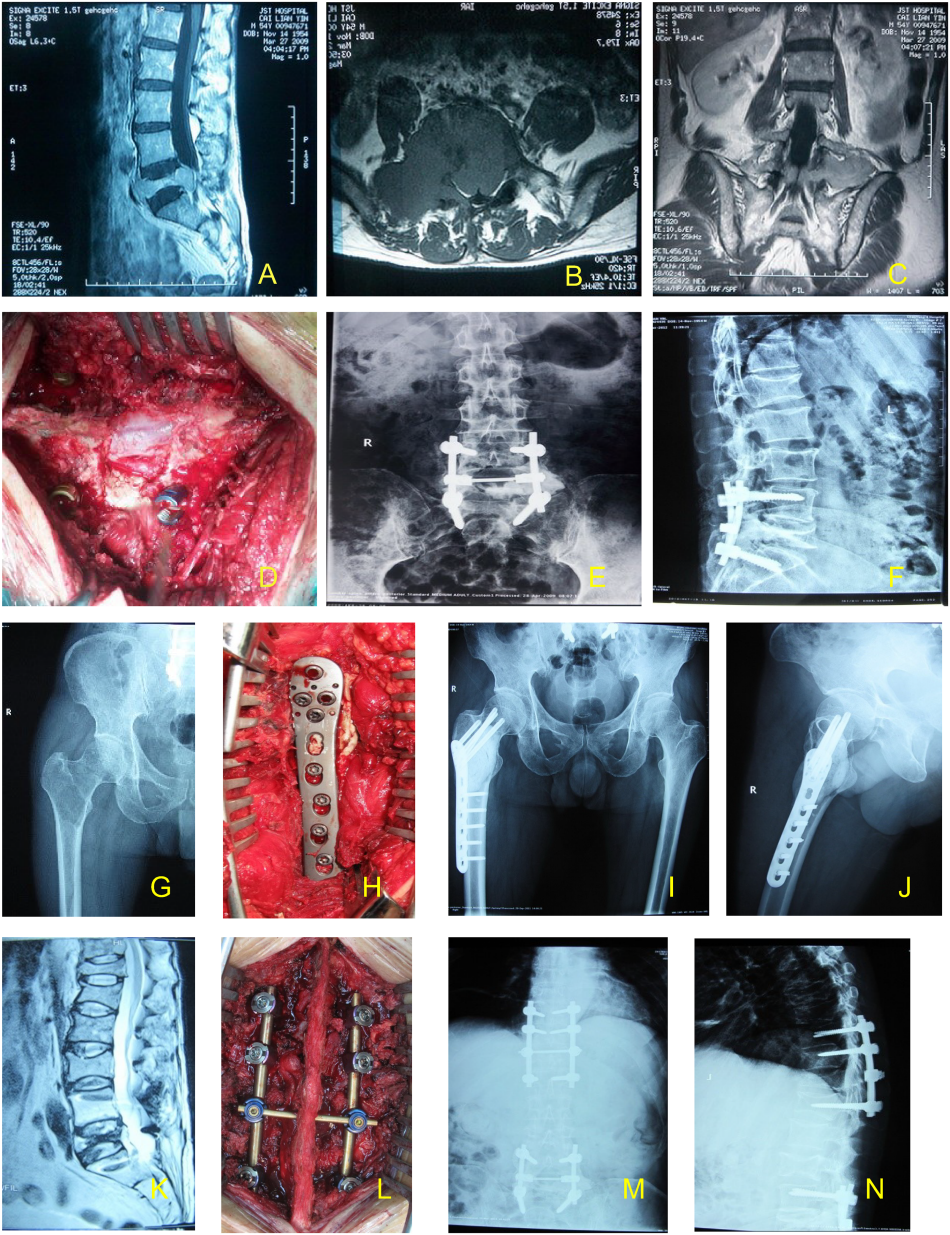
The case of a patient undergoing triple sugery. The first surgery is represented by **(A–F)**. **(A–C)** Prior to surgery, the lesion was located using high-resolution magnetic resonance imaging. **(D)** Surgical field during the first surgery. **(E,F)** Following surgery, the lesion was located by high-resolution magnetic resonance imaging. The second surgery is represented by **(G–J)**. **(G)** MRI of patient prior to the second surgery. **(H)** Surgical field during the second surgery. **(I,J)** Following the second surgery, the lesion was located by high-resolution magnetic resonance imaging. The third surgery is represented by **(K–N)**. **(K)** MRI of patient prior to the third surgery. **(L)** Surgical field during the third surgery. **(M,N)** Following the third surgery, the lesion was located by high-resolution magnetic resonance imaging. MRI, magnetic resonance imaging.

A 54-year-old man presented with lower back pain accompanied by numbness of both lower limbs for 6 months. Magnetic resonance imaging (MRI) on March 24, 2009 indicated that L5 was destroyed with a compressed nerve root and sac (Figures 6A–C). Puncture biopsy revealed that he had abnormal plasma cells. The following results were obtained: IgA (3,420 mg/L), κ light chain (1,280 mg/L). The bone penetrating plasma cells reached 24.5% in the bone marrow and the diagnosis was MM. The patient exhibited the following results on diagnosis: IgA/κtype, DS: III A stage; ISS: stage II MM. The patient received chemotherapy for 2 weeks (bortezomib, dexamethasone and epirubicin) and subsequently he underwent surgery (Figures 6D–F). Chemotherapy was continued 2 weeks following the operation.

After 28 months, the patient reported pain in the right hip while walking. The computed tomography scan indicated destruction in the right intertrochanteric region, with a Mirels' score of 11. The second operation was performed on September 7, 2011, with tumor removal, bone cement filling, titanium plate and screw internal fixation (Figures 6G–J). Following the operation, the pain was mitigated and the patient was able to walk with crutches.

Following 48 months after the first operation, the patient reported severe pain in the chest and upper back, with numb feet and normal movement of the limbs. The second operation was normal. MRI indicated compression fracture in the T11–12 segments. The third operation was performed on March 4, 2013, which alleviated the pain and chemotherapy was continued (Figures 5K–N) until his death in October 2013. The OS and PS were 55 and 54 months, respectively.

## Discussion

Bone disease is a key feature of myeloma, severely affecting quality of life and survival. Drugs like bortezomib and denosumab offer hope in reducing the impact of osteolytic bone disease (OBD) (23). Myeloma-associated osteolytic lesions persist, even in long-term remission (24). Bisphosphonates have been the standard treatment for MMBD, with denosumab, a monoclonal antibody that blocks osteoclast activation, serving as an alternative. Radiotherapy, often used for pain relief, also plays a key role in MMBD management. Few studies have explored the efficacy of surgery for MMBD (23). The main goal in treating MMBD is to alleviate symptoms, including pain, and improve survival and quality of life. Surgery is typically recommended only for patients with pathological fractures, spinal cord or nerve root compression, or lytic bone lesions (25, 26). OBD surgery relieves pain, restores bone continuity, and improves spinal stability, helping patients manage their disease. However, due to the limited sample size of MMBD patients who have undergone surgery, clinical studies cannot provide enough data on its impact on survival and quality of life. The present study demonstrated that surgical can prolong the OS of patients and improve 3-year and 5-year survival rates. This may be in part related to the reduction of pain and other complications as well as the improvement of general quality of life. In the previous research studies conducted by our team, it was found that PKP alone can relieve symptoms, although it could not prolong the OS time of the patients. This may be because open surgery removes more tumor tissue, making it more effective in reducing tumor load. In addition, the decompression achieved with open surgery is more thorough than with PKP/PVP, which are both minimally invasive surgical procedures used to reduce pain caused by vertebral compression fractures in patients with myeloma. In summary, PKP/PVP can be effective in relieving pain, but cannot effectively remove tumor tissue. Therefore, for patients suffering from myeloma bone disease with surgical indications, prompt surgery can provide apparent benefits.

Patients with MM are often accepted and treated by the hematology department (11). Chemotherapy is key in treating MMBD, but some patients initially undergo surgery for pathological fractures, spinal cord/nerve root compression, or soft tissue extramedullary plasmacytoma. As a result, certain patients receive chemotherapy prior to surgery and several patients receive surgery prior to chemotherapy (27, 28). However, there is a lack of information on which order is more beneficial for patients with MMBD. The results of the data of the present study indicate that although certain differences have been noted in the total survival time, no significant differences were reported. Surgery can improve prognosis and alleviate the progression of MM (29, 30). In clinical practice, newly diagnosed patients with surgical indications will first undergo surgery; otherwise, they start with chemotherapy. If surgical indications arise during chemotherapy, surgery is performed followed by continued chemotherapy or other treatments. Surgical indications include instability and pathological fractures, spinal lesions with nerve compression, intractable pain at sites matching MMBD, and extramedullary plasmacytoma in soft tissue (31, 32). The clinical presentation consists of pathological fracture or impending fracture in the long bones of the extremities. Xie et al. (30) verified that radiotherapy in combination with surgery may result in lessened progression of MM for younger patients with solitary plasmacytoma of the spine. This suggests that these patients may benefit more from operative treatment (33, 34).

This study found that patients with MMBD who underwent multiple surgeries had longer OS times and higher survival rates, though surgery count was not significantly linked to prognosis. Multiple surgeries can relieve pain, improve quality of life, and extend OS time, possibly due to longer survival allowing more time for bone-related issues to develop. Therefore, prompt surgery for patients with MM may be an important means to prolong their survival. The reason may be that patients who have undergone one operation exhibit only one indication, while patients who have undergone multiple operations have multiple indications.

Surgical intervention can involve open surgery or be less invasive and can be performed either alone or in combination with other surgeries to maximize the immediate advantage for patients with MMBD. Indeed, prognosis of patients with MM undergoing surgery depends on various factors, including disease progression, postoperative therapeutic course and the potential approach for surgical intervention. Ultimately, the main objectives of MMBD treatment are pain release, long-term neurological recovery and the enhancement in the quality of life and patient survival (14, 35, 36).

Some studies have shown poor consistency between ISS staging and DS staging. DS staging places more emphasis on factors such as serum calcium concentration, dietary structure, and renal impairment, while ISS staging encompasses concise, comprehensive, and more favorable prognostic indicators. Therefore, ISS staging is a recommended method for staging MM. The latest research shown R2-ISS is a straightforward prognostic staging system that enhances the stratification of patients with intermediate-risk newly diagnosed multiple myeloma (NDMM). Its additive approach opens up opportunities for incorporating new prognostic variables in the future (37, 38).

The present study is limited by the fact that it is a retrospective study with a small sample size. Further studies will be required to see if the findings are applicable in larger cohorts. The absence of performance status and high-risk chromosomes information constitutes a limitation of this study. In future observational studies, we will incorporate more detailed clinical information to enhance our analysis capabilities.

## Data Availability

The original contributions presented in the study are included in the article/Supplementary Material, further inquiries can be directed to the corresponding author.
